# Guided Tissue Regeneration Treatment Yields Better Results in Class II Furcations in the Mandible Than in the Maxilla: A Retrospective Study

**DOI:** 10.3390/ijerph18147447

**Published:** 2021-07-13

**Authors:** Odontuya Dorj, Wei-Fang Lee, Eisner Salamanca, Yu-Hwa Pan, Yi-Fan Wu, Yung-Szu Hsu, Jerry C. Y. Lin, Yu-De Lin, Cheuk-Sing Choy, Wei-Jen Chang

**Affiliations:** 1 School of Dentistry, College of Oral Medicine, Taipei Medical University, Taipei 10692, Taiwan; dorj.odontuya@gmail.com (O.D.); shalom.dc@msa.hinet.net (Y.-H.P.); yfwu@tmu.edu.tw (Y.-F.W.); nm8346@yahoo.com.tw (Y.-S.H.); drjerrylin@gmail.com (J.C.Y.L.); 2 Department of Dental Technology and Dental Hygiene, School of Dentistry, Mongolian National University of Medical Sciences, Ulaanbaatar 14210, Mongolia; 3 School of Dental Technology, College of Oral Medicine, Taipei Medical University, Taipei 10692, Taiwan; weiwei@tmu.edu.tw; 4 Department of Dentistry, Chang Gung Memorial Hospital, Taipei 83301, Taiwan; 5 Graduate Institute of Dental & Craniofacial Science, Chang Gung University, Taoyuan 33305, Taiwan; 6 School of Dentistry, College of Medicine, China Medical University, Taichung 40402, Taiwan; 7 Department of Oral Medicine, Infection and Immunity, Harvard School of Dental Medicine, Boston, MA 02115, USA; 8 Sunmax Biotech Co., Ltd., Tainan 744093, Taiwan; lud32@msn.com; 9 Department of Community Medicine, En Chu Kong Hospital, New Taipei City 237, Taiwan; prof.choy@gmail.com; 10 Department of Nursing, Yuanpei University of Medical Technology, Hsin Chu, Taipei 300, Taiwan; 11 Dental Department, Taipei Medical University, Shuang-Ho Hospital, New Taipei City 23561, Taiwan

**Keywords:** guided tissue regeneration, collagen membrane, furcation involvement, upper jaw, lower jaw

## Abstract

Absorbable porcine collagen membrane with a bovine bone graft can be considered for regenerative treatment in periodontal class II furcation defects. We evaluated the clinical efficacy of guided tissue regeneration (GTR) treatment with bovine bone xenograft and a porcine collagen membrane in molars with class II furcations. Probing depth (PD), clinical attachment level (CAL), and bone level (BL) were recorded at baseline and at 3, 6, and 9 months postoperatively. Thirty class II furcation defects from the lower and upper molars were assessed. Significant improvements in PD and CAL were observed from baseline to 9 months in all groups (*p* < 0.01). BL improved in all groups except group A in the upper molars in radiographic assessment (*p* < 0.05). The lower and upper molars showed PD reduction of 50.5% ± 7.44% and 46.2% ± 11.2%, respectively, at 9 months (*p* = 0.044). In furcations of 1–3 mm, the lower and upper molars showed PD reductions of 51.2% ± 4.49% and 36.5% ± 16.14%, respectively (*p* = 0.035). The lower and upper molars showed a CAL gain of 51.1% ± 4.64% and 33.6% ± 18.8%, respectively (*p* = 0.037). Thus, GTR with bovine bone graft and porcine collagen membrane yielded good results in class II furcations, with better results in the lower than in the upper molars.

## 1. Introduction

Uncontrolled periodontitis can lead to loss of bone and attachment and even tooth loss [1]. In some instances, chronic periodontitis can create a periodontal furcation defect in multi-rooted teeth, representing alveolar bone destruction that reaches the area located at the root’s divergence [2]. A recent systematic review indicated that furcation involvement doubles the risk of tooth loss, particularly for first and second molars up to 10–15 years that are maintained in supportive periodontal therapy. The review also reported an association between the degree of furcation defect and tooth loss [2]. Most previous studies mainly employed horizontal measurements for furcation classification [3]. One of the most common classes is class II, in which the periodontal probe is able to enter approximately one-third of the tooth width [3].

Various types of regenerative treatments have been proposed to treat furcation-involved molar teeth, such as guided tissue regeneration (GTR) and guided bone regeneration, using either resorbable or nonresorbable membranes in combination with various types of bone grafts, including autografts, allografts, and xenografts [4]. In molars with furcation involvement, clinical measurements are performed to analyze the remaining periodontal supportive tissues on each root of the furcation-involved area of the multi-rooted tooth. This variable is fundamental for good prognosis [5]. In some cases, the clinical treatment of furcation involvements at the first and second phases of periodontal treatment is unsuccessful [6]. In a clinical study, GTR treatment for class II furcation involvement with bioabsorbable and nonabsorbable expanded polytetrafluoroethylene (e-PTFE) barriers were compared; the outcomes were favorable and similar in terms of the horizontal clinical attachment level (CAL) [7]. In another study, after 6 months of GTR treatment, more favorable horizontal clinical attachment gain outcomes were obtained with biodegradable barriers than with nonresorbable e-PTFE barriers [8]. The resorbable barriers had similar results as the nonresorbable e-PTFE membranes for class II furcation defects and infrabony defects after 12 months postoperatively [9]. These studies suggest that absorbable membranes can be used. Such membranes offer the advantage of no requirement for secondary surgery for removal.

Collagen barrier membranes have shown promising clinical outcomes in the repair of periodontal furcation defects. Type I bovine collagen membranes appear to be an appropriate and efficient material for GTR treatment for class II furcation defects [10]. A recent clinical study evaluated GTR treatment with bovine bone xenograft with or without a collagen membrane for class II furcation defects. It was found that both treatments had comparable clinical effectiveness [11]. A novel porcine collagen membrane used in vivo and in human molars gained considerable attachment, making it a suitable option for improving new bone regeneration in furcation involvement [12,13]. The combined GTR treatment enhanced the vertical probing depth (PD), attachment level gain, and horizontal bone level (BL) gain compared with the resorbable membrane alone [14]. Treatment with mineralized human cancellous allograft with or without collagen membrane enhanced the bone fill in mandibular class II furcations [15]. Moreover, adequate periodontal care for furcation defects can be effective for years [14]; however, the varying anatomy of furcation defects makes the treatment of furcation invasions challenging for clinicians [15]. Studies have reported finite improvement in maxillary molar furcations with GTR treatment; however, these studies did not distinguish between buccal and interproximal maxillary molar furcations [16,17]. Gher et al. reported that most maxillary first molars have a dome shape in terms of the anatomical feature between roots, which complicates treatment. The root concavities initiated near the cementoenamel junction (CEJ) on the buccal and mesial aspects of the tooth root, and it is extended into the furcations of the maxillary molars [18]. Furthermore, they reported the mean root surface area (RSA) in 1-mm increments from the CEJ to the apex for 20 lower first molars. The most prominent mean RSA outcomes were positioned 4–7 mm apical to the CEJ, and 70% of mandibular molars had an intermediate bifurcation ridge. Regarding these anatomical findings, the efficacy of treatment and diagnosis in mandibular first molars may be increased [19]. Knowledge about the particular anatomical features of root furcations is necessary for enhancing the treatment success [20]. Special topographical features of furcations may influence the clinical outcome of the GTR approach in the treatment of furcations, and the membrane, 1–2 mm below the CEJ, cannot secure complete adjustment of furcation defects in the majority of molar teeth [21].

Studies have reported favorable outcomes in mandibular class II furcation defects [22,23], whereas less-promising results were obtained for maxillary furcations [18]. This result can be attributed to their complex trifurcation morphology and the accessibility of furcation defects. A recent systematic review concluded that no gold standard can be established for the regenerative treatment of class II furcation involvement [23]. Therefore, it can be hypothesized that lower molars might improve the clinical outcomes in furcation invasions, and the effect of regenerative treatment in the upper and lower molars remains unclear. Thus, this retrospective study aimed to examine the clinical efficacy of bovine bone xenograft combined with porcine collagen membrane regeneration in three subclasses of class II furcations in the upper and lower molars.

## 2. Materials and Method

### 2.1. Pre-Study Preparation

**Figure 1:**
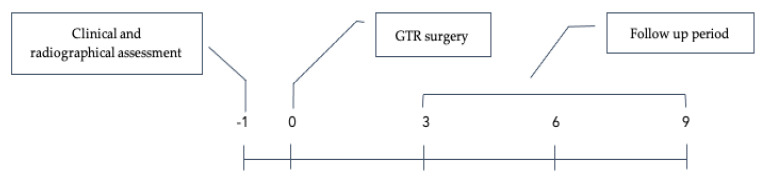
Study timeline (months).

The Ethics Committee and Joint Institutional Review Board of Taiwan approved the study protocol of Taipei Medical University No. 201105001, (this approval was also previously used to publish the pilot results of the present study [13]). A total of 60 class II furcation defects in the upper and lower molars of 60 systematically healthy patients with periodontitis were included in the study after receiving phase I periodontal therapy, which comprised oral hygiene instructions, scaling, and root planning. This study focused on treating class II furcation defects in the maxillary and mandibular molars using GTR with bovine bone xenografts and a porcine collagen membrane with different sizes. The particles ranged from 0.25 mm to 1 mm in size and were procured from Bio-Oss, Geistlich, Germany; membranes with varying sizes, i.e., 1.5 × 2 cm, 2 × 3 cm, and 3 × 4 cm, were procured from Sunmax, Tainan, Taiwan. All hard and soft measurements pre- and post-periodontal therapy were performed by a single periodontist who was blinded to the surgical treatment using a Nabors probe 2N PQ2N7 (Hu-Friedy, Frankfurt am Main, Germany), and a Michigan probe was calibrated at baseline (before treatment) and at 3, 6, and 9 months postoperatively (Figure 1).

### 2.2. Study Design and Patients

This retrospective study included participants who had undergone regenerative treatment with bovine bone xenograft and a porcine collagen membrane for class II furcation defects between 2009 and 2011 at the Department of Periodontology of Taipei Medical University Shuang Ho Hospital, Taipei, Taiwan. All documents and charts of the participants treated with GTR were analyzed carefully by an independent investigator (O.D.). Owing to the study’s retrospective design, data of missing and unresponsive cases were excluded. There were 32 men and 28 women aged 33–73 years (mean age: 50.6 ± 10.6 years) with at least one tooth exhibiting class II furcation defects. The study participants were selected based on the following inclusion and exclusion criteria.

Inclusion criteria:

- Diagnosis of chronic periodontitis;
- Clinical appearance of lingual or vestibular class II furcation defects with >3 mm horizontal width;
- Completion of an oral hygiene therapy comprising oral hygiene guidance, scaling, and root planning;
- O’ Leary plaque control level ≤ 20%;
- At least 2 mm of keratinized gingiva on the furcated teeth.

Exclusion criteria:

- Smoking habit;
- Severe systemic disease;
- Betel nut chewing habits;
- Alcohol consumption;
- Anticoagulant and antibiotic medication for 6 months prior to treatment;
- Poor oral hygiene;
- Pregnant or breastfeeding women;
- Allergy to collagen membrane;
- Lost to follow-up after 3, 6, and 9 months postoperatively.

### 2.3. Surgical Procedure

**Figure 2:**
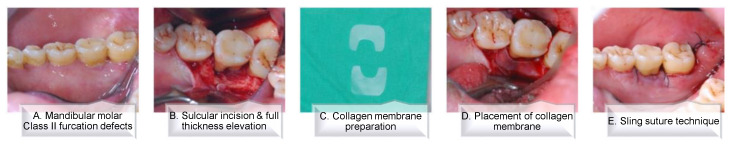
Surgical procedure for regenerative therapy with collagen membrane. (**A**) Mandibular molar class II furcation defects. (**B)** Sulcular incision and full-thickness elevation. (**C**) Collagen membrane preparation. (**D**) Placement of collagen membrane. (**E**) Sling suture technique.

The surgical procedures were previously reported. Briefly, the surgery included sulcular incisions, full-thickness flap elevation, soft tissue debridement, and root planning (Figure 2) [13]. Cervical enamel projections were eliminated using rotary dental instruments. After debridement, bovine bone xenografts were used to fill the furcation defects, and porcine collagen membranes were trimmed in different sizes to completely cover the furcations. A sling suture technique was used to cover both the furcation entrance and the bone graft entirely with a collagen membrane and overlapped the adjoining alveolar crest by 4–5 mm. The periodontal dressing and flap closure sutures were removed 10–14 days after the placement of the dressing, and amoxicillin (500 mg) was administered three times daily for 5 days. The patients were followed up monthly for prevention purposes until 9 months postoperatively.

### 2.4. Data Collection and Measurements

The following details were obtained from all participants: basic characteristics (age and sex), location of the treated furcation defect (upper and lower jaws), clinical parameters of furcation defects (PD and CAL), follow-up time (baseline and 3, 6, and 9 months postoperatively), and postoperative patients’ radiographs. Using a graduated periodontal probe, PD assessments (in millimeters) were performed from the gingival margin to the base of the pocket. The measurements were divided into three groups: A, B, and C. Group A had a depth of 1–3 mm, B: 4–6 mm, and C: >6 mm [24]. CAL was measured from the CEJ to the free gingival margin. BL was calculated vertically from the roof of the furcation to the alveolar crest. Radiographic measurements were assessed using two-dimensional digital radiographic examination by EZ Dental from Asahi Co., Ltd. (Tokyo, Japan) at baseline and at 3, 6, and 9 months postoperatively. BL was also classified into group A, with a depth of 1–3 mm, group B: 4–6 mm, and group C: >6 mm. PD measurements were used as the main guide for furcation classification.

### 2.5. Study Outcomes

The clinical (PD and CAL) and radiographic (BL) changes from baseline to the 3-, 6-, and 9-month postoperative appointments were analyzed, and the corresponding differences between the upper and lower jaws were compared at all time points.

### 2.6. Statistical Analyses

Descriptive statistics were used for presenting the baseline data as means and standard deviations. The changes in both clinical and radiographic parameters from baseline to the 3-, 6-, and 9-month outcomes were evaluated using the two-sample *t*-test. Additionally, the independent *t*-test was used to compare the results between the upper and lower jaws for all the groups. Statistical analyses were performed using Microsoft Excel^®^ software and analyzed using Microsoft Excel^®^ Data analysis tools. At all stages of assessment, a *p* value of <0.05 was considered significant.

## 3. Results

### 3.1. Study Population and Baseline Characteristics

We enrolled 60 patients, including 32 (53.3%) men and 28 (46.7%) women, with a mean age of 50.6 ± 10.6 years. Thirty molars with class II furcation defects in the upper jaw and 30 in the lower jaw were assessed and treated using GTR with bovine bone xenograft and a porcine collagen membrane. All 60 patients were examined at 3, 6, and 9 months postoperatively. The demographic characteristics of the participants and grouping as per PD and study parameters are shown in Table 1.

### 3.2. Clinical and Radiographic Measurements

The analyses of PD and CAL are shown in Table 2. Clinically, the upper molars exhibited a significant improvement in PD in all the groups (group A: 1–3 mm, B: 4–6 mm, and C: >7 mm). The mean reduction values were 1.24 ± 0.55 mm, 2.21 ± 0.40 mm, and 3.84 ± 0.88 mm, respectively, for the groups in the upper jaw after 9 months, and the corresponding values were 1.77 ± 0.13 mm, 2.38 ± 0.62 mm, and 3.73 ± 0.49 mm, respectively, for the groups in the lower jaw (Table 2). There were significant differences between the time points at baseline and at 9 months postoperatively (*p* < 0.01). Moreover, a notable improvement in CAL was observed in all the groups in the upper jaw (1.21 ± 0.72 mm for A, 2.51 ± 0.82 mm for B, and 3.70 ± 0.57 mm for C; *p* < 0.001). Similarly, at 9 months after GTR treatment with bovine bone xenografts and a porcine collagen membrane, a significant recovery in CAL was observed in all the groups in the lower jaw (2.15 ± 0.58 mm for A, 2.33 ± 0.74 mm for B, and 3.32 ± 1.22 mm for C; *p* < 0.001).

**Figure 3:**
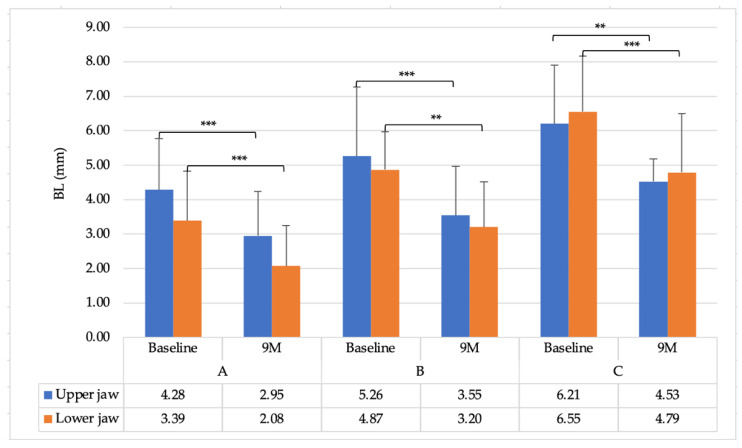
Radiographic parameters of the upper and lower jaws (Group A: 1–3 mm; B: 4–6 mm; and C: >6 mm). * *p* < 0.05 and ** *p* < 0.01 *** *p* < 0.001, respectively.

**Figure 4:**
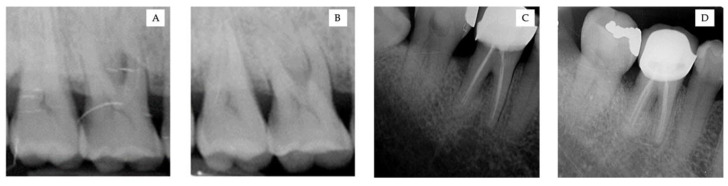
(**A**,**B**) Radiograph of the upper right molar. (**C**,**D**) Radiograph of the lower left molar. (**A**–**C**). Before GTR treatment. (**B**–**D**). 9 months after treatment with bovine bone xenograft and a porcine collagen membrane.

The evaluation and radiograph of BL are shown in Figure 3 and Figure 4, respectively. Radiographically, there were no significant changes among the time points in group A in the upper jaw. However, there was a 1.31 ± 0.59 mm change in the lower jaw after 9 months, which was significant (*p* < 0.05). However, the mean changes in group B were 1.71 ± 0.59 mm and 1.67 ± 0.21 mm for the upper and lower jaw, respectively. There were significant differences at different time points at baseline and 9 months in both the upper and lower jaws (*p* < 0.01). Furthermore, group C exhibited a considerable difference at 9 months postoperatively, with 1.68 ± 1.03 mm and 1.76 ± 0.08 mm for the upper and lower jaws, respectively (*p* < 0.05).

### 3.3. Comparison between the Upper and Lower Jaws

**Figure 5:**
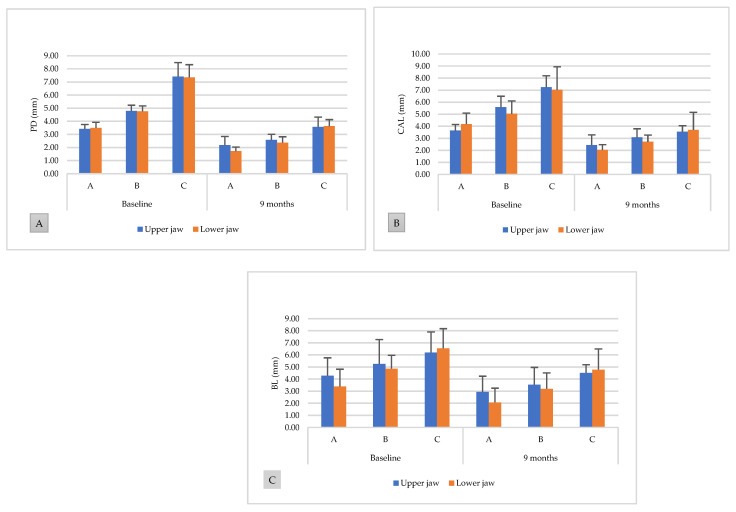
Comparison of hard and soft tissue levels between the upper and lower jaws at baseline and 9 months postoperatively. (**A**) Probing depth, (**B**) clinical attachment level, and (**C**) bone level.

The comparative mean values of hard and soft tissues are shown in Figure 5. There were no significant differences between the groups at all time points in both the upper and lower jaws. However, both the upper and lower jaws showed comparable reduction and gain tendency at 9 months after GTR treatment.

The mean percentage of hard tissue gain at different time points for the upper and lower jaws is summarized in Table 3. Overall, a similar tendency was observed in BL gain in all the groups (A: 1–3 mm, B: 4–6 mm, and C: >6 mm) at all time points, with no significance in intragroup evaluations, except in group A in the lower jaw (*p* < 0.05). However, no significant differences were detected at any time point in the intergroup assessments (*p* > 0.05).

**Figure 6:**
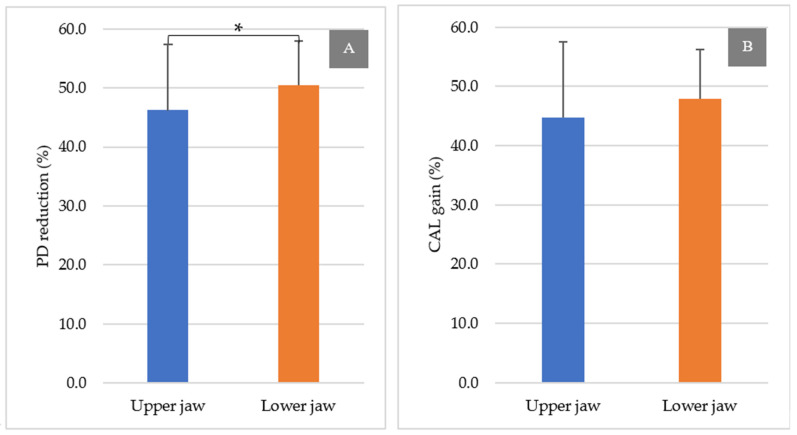
PD reduction and CAL gain at 9 months postoperatively in the upper and lower jaws (%). (**A**)**:** PD reduction at 9 months and (**B**)**:** CAL gain at 9 months. * *p* < 0.05.

Furthermore, the mean percentage of soft tissue reduction at 9 months postoperatively for the upper and lower jaws is illustrated in Figure 6. The lower molars exhibited a significantly higher PD reduction than the upper molars at 9 months postoperatively (upper jaw: 46.2% ± 11.2%, lower jaw: 50.5% ± 7.44%, *p* = 0.044). However, CAL gain failed to show a significant difference between the upper and lower jaws (upper jaw: 44.8% ± 12.7%, lower jaw: 47.9% ± 8.32%, *p* = 0.131).

In particular, 9 months after GTR, group A revealed a significant PD reduction in the lower jaw compared with that in the upper jaw (upper jaw: 36.5% ± 16.14%, lower jaw: 51.2% ± 4.49%, *p* = 0.035). Similarly, the CAL gain was significantly different between the lower jaw and upper jaw in group A (upper jaw: 33.6% ± 18.8%, lower jaw: 51.1% ± 4.64%, *p* = 0.037). However, no significant differences were noted at all follow-ups between the upper and lower jaws in groups B and C (figures not shown).

## 4. Discussion

**Figure 7:**
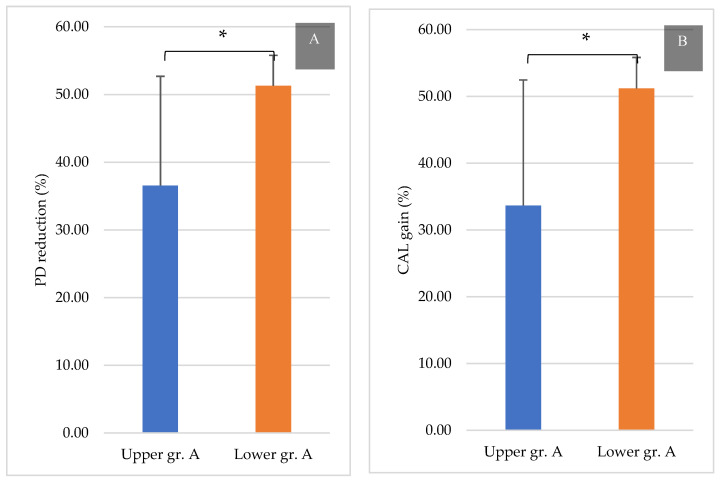
PD reduction and CAL gain at 9 months post-treatment in group A (%). (**A**): PD reduction at 9 months in group A and (**B**): CAL gain at 9 months in group A. * *p* < 0.05.

In the present study, a combination of bovine bone xenograft and a porcine collagen membrane was used to treat furcation defects in the upper and lower jaws. The results showed more improvement in PD, CAL, and BL in the lower molars than the upper molars furcation involvement after GTR treatment (Figure 7). Although the overall radiographic and clinical results improved considerably in both the jaws, there was limited statistical significance of the results between the groups. GTR using bovine bone xenografts and a porcine collagen membrane can be considered a safe treatment for cases of furcation involvement, providing equally favorable outcomes for upper and lower molar furcation. However, better results were observed in furcations with 1–3-mm vertical involvement.

The reduction in pocket depth is the primary goal of periodontal treatment. In the present study, the PD in the upper and lower jaws in subgroup A was reduced from 3.42 mm and 3.49 mm at baseline to 2.18 mm and 1.71 mm at 9 months after treatment, with a mean PD reduction of 1. 24 mm and 1.77 mm, respectively. A recent study reported similar results with a probing pocket depth reduction of 1.3 mm after 6 months of therapy using demineralized freeze-dried bone allograft (DFDBA) bone graft and a bioabsorbable membrane [25]. Another study reported a PD reduction of 2.4 mm after xenograft alone vs. an anorganic bovine bone xenograft and a bioabsorbable collagen membrane at 6 months [26]. This reduction in PD, which was better than that achieved in our study, could be attributed to the smaller sample size in their study. Moreover, the PD reduction in group A was similar to that reported previously, in which the PD reduction varied from 1.7 mm and 0.9 mm [16] and 1.6 mm and 1.3 mm [27] to 2.8 mm and 1.6 mm [9] in the test and control groups, respectively, at 6 months. In our study, at 9 months, more significant PD reduction was reported in groups B (upper: 2.21 mm and lower 2.38 mm) and C (upper: 3.84 mm and lower 3.73 mm); however, the between-group differences were not significant.

The CAL gain in group A between the upper and lower molars after 9 months was significant. These results are similar to those reported by Taheri et al. [26] and Houser et al. [28]. These studies involved 18 and 31 class II furcations, respectively, and performed follow-up for 6 months. The studies reported a CAL gain of 1.8 mm using bovine bone graft and membrane. In our study, furcation class II with vertical reduction of 1–3 mm showed CAL gains of 2.15 mm and 1.21 mm in the lower and upper molars, respectively. Metzler et al. [16] also demonstrated lower CAL gain for the maxillary areas, similar to the results of Pontoriero et al. [27], who reported CAL gains of 0.7 mm and 0.1 mm in the same location. These studies correlated this difference between the upper and lower molars to the complex morphology and accessibility of furcations. Other researchers investigating GTR treatment for buccal furcation defects showed that the treatment efficacy was improved by enhancing the clinical attachment and BL gain and reducing soft tissue recession [27]. Other studies with longer follow-up periods showed a CAL gain of 1.33 mm at 12 months after GTR treatment with e-PTFE membranes combined with DFDBA [29]. The CAL gain was lower than that in groups B (upper: 2.51 mm and lower 2.32 mm) and C (upper: 3.70 mm and lower 3.32 mm) in the present study. Moreover, other studies reported less CAL gain using only biodegradable membranes after 6 months [8], A CAL gain of 1.67-mm after 12 months of GTR treatment using collagen membrane alone in mandibular molars with class II furcation involvement [30], and a mean CAL change of 1.8 mm after GTR with bovine bone graft and membrane [28]. These results of CAL gain in previous studies were lower than the current finding of 2.15 mm in subclass A in the lower jaw. Furthermore, a significant gain in CAL between the upper and lower molars was observed in group A in this study. According to Majzoub et al., the mean CAL gain was close to that in our study after 1 year of GTR treatment, which was significantly correlated with the vertical component and location of the furcations [31]. The same researchers demonstrated that buccal furcation defects of the lower molars showed a CAL gain of 2.35 mm, which was similar to that in group B in the lower jaw in the present study (Figure 7). Both vertical and horizontal CAL gain ranged from 0.2 to 4.0 mm in the study by Karring et al. [32], while CAL gain ranged from 1.21 to 3.70 mm in all groups in the present study. Some studies have indicated that factors such as bone defect anatomy, root morphology, plaque, and oral hygiene control can modify these changes in the CAL [29,33,34].

In terms of bone level, at 9-months after treatment, the average BL change was 1.6 mm in the lower and upper jaws. Houser et al. [28] found 2.0 mm changes in the hard tissue measurements at 6 months. A clinical trial by Pruthi et al. [35] demonstrated the effectiveness of a bioabsorbable collagen membrane and e-PTFE using two lower molars of 17 patients; however, the vertical bone fill of the lower molar was 1 mm with e-PTFE and 0.81 mm with the collagen membrane after 12 months. These results were inferior to our findings and could be attributable to the smaller sample size in the previous study [35]. Taheri et al. reported a gain of 1 mm with open flap debridement (OFD) vs. 1.9 mm bone filled while combining bovine bone graft with collagen membrane, after 6 months [26]. This outcome was 0.3 mm better than our BL gain; however, our results were better than the result for their control. These results indicate that regenerative treatment for class II furcations is better than OFD alone. Different treatment modalities with bone replacement graft are associated with a better outcome [23].

The present results show that GTR treatment using bovine bone xenograft and a bioabsorbable porcine collagen membrane provides better clinical results in the upper and lower molars. Both the jaws showed significant PD reduction and CAL and BL gain from baseline to 9 months. Moreover, the present study demonstrated a favorable outcome in the lower molars in terms of overall PD reduction and CAL gain after 9 months of treatment. Specifically, group A showed a more significant PD reduction and CAL gain in the lower molars than in the upper molars. Whilst treating the patients involve in this study it was found that this approach has a fast, easy learning curve and a high degree of success for a common treatment done by periodontists and dentists on a daily basis, while it could incur low costs for future patients. The bigger the furcation involvements are, the more difficult it is to keep free from pathogenic bacteria. This regenerative treatment can be used in any furcation involvement to reduce its size and help patients to improve their oral hygiene, while professionals doing the treatment can have a better understanding of the regeneration level that can be achieved after surgery in upper and lower molars.

It is crucial to consider some limitations that might affect the significance of the statistical analyses. The small sample size may restrict the ability to generalize our results. Due to the retrospective nature of the study, the influence of oral hygiene status on the GTR treatment, such as plaque index and full-mouth bleeding, could not be examined. Despite the promising clinical results of our study, biological periodontal regeneration cannot be determined via clinical evaluation. Finally, as per the research protocol, the follow-up period was limited to 3–9 months. Accordingly, studies are needed to demonstrate the efficacy of bovine bone graft and porcine collagen membrane treatment for a longer period while using more accurate assessment techniques, such as cone beam computed tomography or dental magnetic resonance imaging and different treatment modalities in the upper and lower jaws.

## 5. Conclusions

This study showed that GTR treatment with bovine bone xenograft using a porcine collagen membrane yielded slightly better PD reduction and CAL gain in lower molar class II furcation defects with 1–3 mm vertical bone loss than in the upper molars. Overall, the combined GTR treatment yielded a good clinical outcome in the upper and lower molars by increasing BL and CAL, while reducing the probing pocket depth, making the treatment a possible therapy for class II furcation involvements.

## Figures and Tables

**Table 1:** Demographic characteristics of the participants according to the upper and lower jaws.

Baseline Characteristics	Upper Jaw (*n* = 30)	Lower Jaw (*n* = 30)
Demographic data		
Male (***n***, %)	20, 66.6%	12, 40%
Female (***n***, %)	10, 33.3%	18, 60%
Age, mean ± SD	50.03 ± 10.96	51.10 ± 10.38
Grouping by PD (***n***, %)		
A (1–3 mm)	6, 20.0%	9, 30.0%
B (4–6 mm)	13, 43.3%	13, 43.3%
C (>6 mm)	11, 36.6%	8, 26.6%
Parameters, mean ± SD (mm)		
BL	5.41 ± 1.88	4.87 ± 1.78
PD	5.46 ± 1.74	5.07 ± 1.61
CAL	5.80 ± 1.57	5.31 ± 1.69

BL, bone level; PD, probing depth; CAL, clinical attachment level; SD, standard deviation.

**Table 2:** Clinical parameters in the upper and lower jaw according to groups (A, B, and C).

Groups	PD			CAL		
	Baseline vs. 9 Months		*p*	Baseline vs. 9 Months		*p*
Upper jaw						
A	3.42 ± 0.34	2.18 ± 0.66	<0.01	3.64 ± 0.51	2.43 ± 0.85	<0.01
B	4.78 ± 0.45	2.57 ± 0.44	<0.001	5.59 ± 0.90	3.08 ± 0.71	<0.001
C	7.40 ± 1.08	3.56 ± 0.76	<0.001	7.24 ± 0.96	3.54 ± 0.51	<0.001
Lower jaw						
A	3.49 ± 0.44	1.71 ± 0.32	<0.001	4.18 ± 0.92	2.03 ± 0.44	<0.001
B	4.75 ± 0.42	2.37 ± 0.45	<0.001	5.03 ± 1.07	2.71 ± 0.57	<0.001
C	7.35 ± 0.97	3.62 ± 0.50	<0.001	7.02 ± 1.92	3.70 ± 1.46	<0.001

PD, probing depth; CAL, clinical attachment level. Group A: 1–3 mm; B: 4–6 mm; and C: >6 mm.

**Table 3:** Percentage of BL gain in the upper and lower jaw groups at different time points.

Groups		Bone Level Gain (%)				
		3 Months	6 Months	*p*	9 Months	*p*
				3 vs. 6 Months		3 vs. 9 Months
Upper jaw	A	28.7 ± 7.78	31.1 ± 8.20	0.113	32.9 ± 8.54	0.022
	B	24.3 ± 17.8	28.0 ± 15.1	0.363	32.3 ± 16.1	0.100
	C	13.7 ± 15.8	20.9 ± 21.4	0.146	23.0 ± 20.8	0.812
Lower jaw	A	27.6 ± 13.3	36.8 ± 15.5	0.022	41.0 ± 14.2	0.003
	B	28.2 ± 16.4	30.5 ± 22.2	0.428	33.3 ± 23.2	0.127
	C	20.3 ± 9.15	25.5 ± 8.65	0.101	28.8 ± 11.9	0.082

Group A: 1–3 mm; B: 4–6 mm; and C: >6 mm.
